# Provider perspectives on barriers and facilitators to engaging with caregivers of older, rural patients

**DOI:** 10.1093/geroni/igaf122.2769

**Published:** 2025-12-31

**Authors:** Eileen Dryden, Aileen McGrory, Jacqueline Boudreau, Lynette Kelley, Marianne Desir

**Affiliations:** Veterans Health Administration, Bedford, Massachusetts, United States; Veterans Health Administration, Bedford, Massachusetts, United States; Veterans Health Administration, Bedford, Massachusetts, United States; Veterans Health Administration, Aurora, Colorado, United States; Veterans Health Administration, Miami, Florida, United States

## Abstract

Over 50 million adults in the US alone are caregivers. Caregivers and the people they care for experience better health outcomes when caregivers are actively engaged with and supported by care recipients’ healthcare teams. Too often, these informal caregivers (e.g. caregivers of spouses, children, friends) remain unacknowledged by the healthcare system, unengaged with care teams, and disconnected from needed support services. This can be particularly challenging for rural caregivers who more frequently report ‘having no choice in taking on care’ and more difficulty finding affordable services in their area and providing more hours of care per week compared with their suburban and urban counterparts. We interviewed 27 Veterans Health Administration providers about barriers and facilitators to identifying and engaging with caregivers of older, rural patients. Providers universally identified barriers to providing support to caregivers, such as lack of time and perceived scarcity of resources, and felt that more support for caregivers was needed. Many felt privacy policies limited their speaking with caregivers. Those who already discuss caregiver needs had developed their own, mostly unstandardized methods for recognizing caregivers who are struggling, initiating private time with caregivers, or asking questions to elicit needs. We highlight these provider perceptions--including how they identify caregivers, how caregiver needs and distress are determined, and systems-level barriers to information sharing--and discuss how these may be addressed in a research-informed intervention.

